# Patient Embeddings From Diagnosis Codes for Health Care Prediction Tasks: Pat2Vec Machine Learning Framework

**DOI:** 10.2196/40755

**Published:** 2023-04-21

**Authors:** Edgar Steiger, Lars Eric Kroll

**Affiliations:** 1 Zi Data Science Lab Department IT and Data Science Central Research Institute of Ambulatory Health Care in Germany (Zi) Berlin Germany

**Keywords:** electronic health records, ICD, machine learning, health care, data, diagnosis, model, drug, drug prescription, performance, applications, quality, prevention

## Abstract

**Background:**

In health care, diagnosis codes in claims data and electronic health records (EHRs) play an important role in data-driven decision making. Any analysis that uses a patient’s diagnosis codes to predict future outcomes or describe morbidity requires a numerical representation of this diagnosis profile made up of string-based diagnosis codes. These numerical representations are especially important for machine learning models. Most commonly, binary-encoded representations have been used, usually for a subset of diagnoses. In real-world health care applications, several issues arise: patient profiles show high variability even when the underlying diseases are the same, they may have gaps and not contain all available information, and a large number of appropriate diagnoses must be considered.

**Objective:**

We herein present Pat2Vec, a self-supervised machine learning framework inspired by neural network–based natural language processing that embeds complete diagnosis profiles into a small real-valued numerical vector.

**Methods:**

Based on German outpatient claims data with diagnosis codes according to the International Statistical Classification of Diseases and Related Health Problems, 10th Revision (ICD-10), we discovered an optimal vectorization embedding model for patient diagnosis profiles with Bayesian optimization for the hyperparameters. The calibration process ensured a robust embedding model for health care–relevant tasks by aggregating the metrics of different regression and classification tasks using different machine learning algorithms (linear and logistic regression as well as gradient-boosted trees). The models were tested against a baseline model that binary encodes the most common diagnoses. The study used diagnosis profiles and supplementary data from more than 10 million patients from 2016 to 2019 and was based on the largest German ambulatory claims data set. To describe subpopulations in health care, we identified clusters (via density-based clustering) and visualized patient vectors in 2D (via dimensionality reduction with uniform manifold approximation). Furthermore, we applied our vectorization model to predict prospective drug prescription costs based on patients’ diagnoses.

**Results:**

Our final models outperform the baseline model (binary encoding) with equal dimensions. They are more robust to missing data and show large performance gains, particularly in lower dimensions, demonstrating the embedding model’s compression of nonlinear information. In the future, other sources of health care data can be integrated into the current diagnosis-based framework. Other researchers can apply our publicly shared embedding model to their own diagnosis data.

**Conclusions:**

We envision a wide range of applications for Pat2Vec that will improve health care quality, including personalized prevention and signal detection in patient surveillance as well as health care resource planning based on subcohorts identified by our data-driven machine learning framework.

## Introduction

Public health surveillance and health care research in many countries depend on electronic health records (EHRs), including claims data [1-4]. In these records, patients’ medical diagnoses are often coded according to a string-based disease classification convention, for example, the International Statistical Classification of Diseases and Related Health Problems, 10th Revision (ICD-10) [5]. Their sequence of ICD codes characterizes the medical history of every patient.

Common tasks in clinical, epidemiological, or health care research on claims data expect numerical input (eg, regression and classification tasks such as linear or logistic regression or advanced machine learning tools such as gradient-boosted trees and deep learning). These methods are often used to predict specific health outcomes [6-17] or the utilization of health care institutions [18-22].

To derive numerical input for these methods from the string-based diagnosis profiles, a procedure called binary encoding (or binarization, one-hot encoding) is most often used [6-11,15-17,20-24]. Using binary encoding, diagnoses are represented numerically by either 1 or 0, if the patient had or did not have the chosen diagnosis, respectively. As the pool of possible diagnostic codes is vast, binary encoding usually relies on a selected subset of diagnoses chosen by either field experts [6,16] or data-driven feature selection [10,15,17]. Diagnoses can also be represented by the number of times they appear [9,12,25,26]. Most often, they are pooled into clinical groups before further analysis [18-22,24,27-29].

Ideally, a disease classification such as ICD-10 would only cover clearly distinguishable medical conditions and concepts, but in reality, we have to deal with overlaps and uncertainties. Therefore, a faithful numerical representation of the patient’s medical history needs to take into account that different ICD codes may represent similar or even identical underlying issues. Frequently, computational and methodological constraints limit the number of diagnoses and interaction effects that can be considered. Binary encoding suffers in this regard, as it considers medical diagnoses as distinctive and unrelated features. As such, it limits the methodical progress of prediction tasks on claims data, especially the application of advanced machine learning methods. Thus, other methods of numerical representation of ICD diagnosis codes should be investigated to enable better individual health care and more precise prediction of health care demand.

We investigate herein how a real-valued numerical representation (or vectorization, embedding) (see Chapter 15 in [30]) of patients’ medical diagnosis profiles that uses their whole diagnostic ICD profiles can be derived. This embedding should compress the information from up to 14,877 possible 5-digit International Statistical Classification of Diseases and Related Health Problems, 10th revision, German Modification (ICD-10-GM) 2019 [31] codes, improve the performance of common health care prediction tasks, and let advanced (nonlinear) machine learning methods reach their full potential when used on claims data.

To find such an embedding, we employ a self-supervised machine learning algorithm inspired by natural language processing (NLP), namely, Doc2Vec [32], which itself is an extension of Word2Vec [33,34]. It has been applied to nonlanguage-specific tasks before [35-37]. Many studies [14,29,38-42] have investigated embeddings of the ICD codes themselves, whereas some [14,25,42] arrived at patient-level embeddings for specific prediction tasks (Supplementary Table S1 in Multimedia Appendix 1). Here, we want to broaden the scope of the possible applications to general health care–related questions. It has been shown that hyperparameter tuning for Word2Vec and Doc2Vec can lead to considerably better results, especially on nonlanguage-related tasks [35,37]. As such, we employ a Bayesian search on a hyperparameter grid to identify an optimal model for the vector embedding procedure. We evaluate our embedding model on broad health care prediction tasks with standard (linear and logistic regression) and advanced machine learning techniques (gradient-boosted trees). We also test how well the vectorization works with smaller data sets and how well it handles missing data with random data dropout sampling. In addition, we inspect the results visually in a 2D projected space along with a clustering of the embedded patient profiles to reveal the properties of our cohort. Finally, we evaluate the resulting vectorization model for the health care–relevant task of predicting drug spending at the patient level.

Our method gave better results than binary encoding, but only after tuning the hyperparameters and on large enough data sets. The compression of the information of thousands of ICD-10 codes into a vector space of no more than 100 dimensions was achieved. We observed large performance gains using gradient-boosted trees with the vector embedding over classic linear or logistic regression with binary-encoded data. In addition, the vectorization models are more robust to missing data than baseline binary encoding. The final model learned on our extensive data can be shared and used by other stakeholders on much smaller data sets (eg, for supervised machine learning methods that predict clinical or other health care outcomes).

## Methods

### Data

The diagnosis data are based on comprehensive nationwide outpatient claims data from 2016 to 2019 of all patients with statutory health insurance (SHI) in Germany. According to the Federal Statistical Office [43], there were 73,009,237 persons eligible for the SHI (87.8% of the population) in 2019. The pseudonymous data include diagnoses for all people in Germany with SHI who visited an outpatient physician in 2016 or later. Among others, the data include demographic characteristics such as age and gender, as well as diagnoses with markers of certainty and other billing-relevant information. These data do not contain information on inpatient treatment in hospitals. Diagnoses are coded according to the ICD-10-GM [31]. In addition to the diagnosis data, we extracted individual information on prescribed and dispensed medications from the pseudonymous data of nationwide outpatient drug prescriptions. The claims data and the prescription data are linked by patient information (compare [44]).

We chose N=11,200,000 patients at random from the full population of people with SHI because technical limitations make it impossible to use the full data. To achieve this study sample size, we shuffled all patients in the claims database randomly and selected the top N records for the sample. All patients with at least one data entry after 2016 were eligible. The sample is divided into 4 data sets by random subsampling from the study population (Textbox 1).

**Figure 1:**
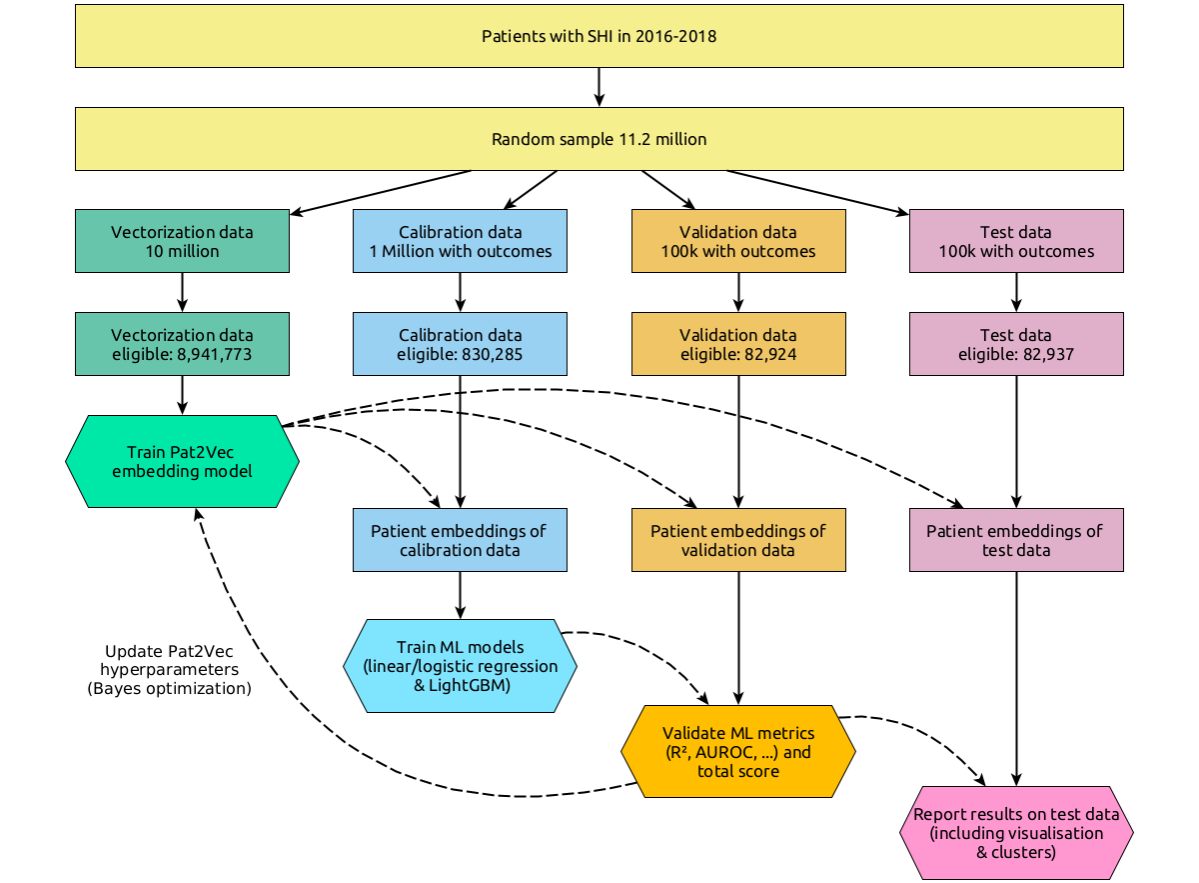
Flowchart of data sampling and algorithmic schematic. Patient data flows are represented by solid, straight lines, while machine learning models and other meta-information flows are represented by dashed, curved lines. Rectangles are patient data, while hexagons are algorithms or analysis methods. AUROC: area under the receiver operating characteristic curve; ML: machine learning; SHI: statutory health insurance.

These samples were filtered for patients with consistent information regarding gender and age during the years considered for analysis (2016 to 2019). The training data in (1) for the vectorization model were restricted to ICD-10 codes (5-digit notation) from 2016 to 2018, whereas the calibration, validation, and test sets in (2)-(4) were restricted to codes from 2018. Only patients with at least one confirmed diagnosis during the period in question were kept. This left us with sample sizes of 8,941,773 (vectorization training), 830,285 (calibration training), 82,924 (validation), and 82,937 (test), see Figure 1.

Because of the regulations of the German health care system (see “The German Health Care System” in [45], or a more detailed description of the German system in [46]), diagnoses are available on a quarterly basis (but without temporal order within a quarter), with reference to cases and places of treatment. As such, we generated a sequence of codes for each patient with a certain temporal order: confirmed diagnoses are grouped by case and place of treatment, and these groups are ordered by temporal succession of quarters, but if more than 1 group appears within one-quarter, these groups are shuffled randomly within the quarter (as well as diagnoses within a group).

Furthermore, when training the model (see below), only diagnoses that were seen at least 100 times in the training data were taken into account.

As health care–relevant outcomes in (2)-(4), we used 4 different quantities for calibration: the *number of cases* (a proxy indicator for the number of medical consultations), (ambulatory) *emergency health care utilization*, *age*, and *gender*. The number of cases in 2019 is approximate due to data limitations: a case is defined as the unique combination of a quarter, a patient, a treating medical facility, the billing association of SHI physicians, and the time stamp of data processing. The binary outcome of emergency health care utilization is 1 if at least one case in 2019 of the respective patient was billed as an emergency, and 0 otherwise. The sociodemographic variables age (in years) and gender (binary-encoded) were also extracted from the data.

As data for robustness analysis against diagnosis dropout, we randomly dropped 10%, 25%, or 50% of diagnosis codes for each patient (rounded to nearest number, but kept at least one code).

As data for robustness analysis against varying training data set sizes, we used different percentages of the original vectorization training data (reducing the vectorization data from 10 million patients to 10,000 patients).

For a further analysis, we extracted the drug prescription costs from the ambulatory drug prescription data of residents in Germany with SHI. These costs are the total (in euros) of all billed prescribed drugs for the respective patient in 2019 (if any, otherwise 0).

**Data sets obtained by random subsampling from the study population.**

1. **Vectorization**
  A total of 10,000,000 patients as a vectorization training set for self-supervised machine learning to learn a model for numerical representation (embedding) of patients’ profiles.
2. **Calibration**
  A total of 1,000,000 patients with embeddings based on a model from (1) serving as a calibration training set for supervised machine learning on prediction tasks.
3. **Validation**
  A total of 100,000 patients with embeddings based on a model from (1) serving as a validation set for the calibration prediction models learned in (2) and, in turn, hyperparameter tuning of vectorization in (1).
4. **Test**
  A total of 100,000 patients as a test set for final analysis and presentation of the results.

### Ethical Considerations

The use of claims data for this analysis is governed by the German Code of Social Law (SGB X 80 in conjunction with SGB V 68c): our study aims to improve health care quality by exploring diagnoses profiles and predicting health care–relevant outcomes. While approval and consent of individual human patients within the cohort are operationally impossible to acquire, they are also not required by the German Code of Social Law as we used deidentified, routinely collected data in a retrospective study. In addition, we argue that the conclusions we can draw from our analyses are in the best interest of patients and will improve future public health services.

### Binary Encoding and Baseline Model

Binary encoding creates a data matrix with rows for patients and columns for variables. Each variable represents one of the diagnoses being looked at (out of a chosen subset of all available diagnoses) and is given a 1 in the corresponding row and column if the patient had that diagnosis and a 0 if they did not.

Here, we employ such a binary encoding approach as a baseline model: First, we sorted all confirmed unique ICD-10 diagnosis codes from 2019 by the number of patients with this diagnosis in the data. Second, for a given number M of top diagnoses and the sample patients from above, we formed the appropriate data matrix with M columns corresponding to the top M diagnoses and each row representing a patient, using binary encoding like described above. This is the baseline model for numerization of the diagnosis codes and will be compared with the real-valued patient-level embedding described in the next section.

### ICD2Vec and Pat2Vec

Similar to [14], we used an advanced approach to a real-valued embedding of diagnosis codes, applying a method from NLP called Word2Vec and its extension Doc2Vec [32-34]. Trained on a corpus of text data, Word2Vec vectorizes individual words and keeps their semantic meaning by mapping similar or related words to similar vectors (according to multidimensional distance measures in a Euclidean space) and antagonistic words to diverging vectors. As an extension to Word2Vec, the Doc2Vec algorithm also learns vectors for each document. Similar documents are represented by vectors that are similar to those of the similar documents.

Word2Vec is in fact a (shallow) neural network in the sense that individual words are represented by vectors (embeddings) of a fixed size, and the entries of these vectors are used directly to predict the vectors of other words in a single-layer neural network; that is, the embeddings are themselves the parameters of the single hidden layer. Word2Vec goes over every word in each document step-by-step and repeatedly during training and updates the neural network’s parameters (or rather, the embeddings) by either predicting from the current word the neighboring or context words as targets (skip-gram) or predicting a target word from the neighboring or context words (continuous bag of words) [33]. In both cases, the update to the network’s parameters after training on a single word would include updating all parameters for all words that are not in the context. For computational efficiency (because of large vocabularies), this is circumvented by either updating only some negative examples of words that are not in the context of the word under consideration [34] or by applying a hierarchical softmax to the network update [33]. In fact, it is also possible to apply both techniques at the same time.

Doc2Vec is an extension to the Word2Vec algorithm in the sense that it is applied in parallel to Word2Vec. Additionally, while learning the vector embeddings of every word in the corpus, the vector embeddings of the documents that form the corpus are learned in the same manner. Doc2Vec can be trained in 2 different ways [32]: either with “distributed memory” (DM; similar to Word2Vec’s continuous bag of words), where each target word from the document is predicted using both the context words and the document’s embedding, or with “distributed bag of words” (DBOW; similar to Word2Vec’s skip-gram), where target words from the document are predicted using the document itself and separately updating the context words.

For more background on neural networks and how they are applied to NLP tasks, see [47] and [48].

In our framework, we treat every ICD-10 diagnosis code as a word and the sequence of diagnosis codes for a patient as a document. These documents are our corpus data for training ICD2Vec (by applying Word2Vec to ICD-10 codes) and Pat2Vec (by applying Doc2Vec to patients’ sequences of diagnosis codes).

For training the 2Vec algorithms, we have to choose a vector size of M (among other parameters; see below). Pat2Vec is trained on the patients’ sample data and then gives us a data matrix with M columns, where each row or patient is a vector of length M (the embedding of the corresponding patient), encoding *all* of their diagnoses. Additionally, we obtain in parallel a vectorization of the ICD-10 codes themselves (Word2Vec/ICD2Vec), where each code is represented by a vector.

### Hyperparameter Tuning

The 2Vec algorithms need several parameters as input for the training of the vectorization model. These are referred to as hyperparameters and have different considered ranges (Textbox 2).

Following previous research [35,37], we tuned the hyperparameters for the vectorization model using a Bayesian hyperparameter optimization [49] over the ranges given above. We calibrated and validated the resulting vectorization models with supervised machine learning (see the next section) using the holdout calibration and validation data on the 4 calibration outcomes.

**Hyperparameters and their ranges.**

1. **Vector size (100)**
  Length of the vector assigned to each patient. We hold this fixed while tuning the hyperparameters, but we will vary this value afterward for comparisons.
2. **Minimal count (100)**
  Only diagnoses that appear at least 100 times in the data are considered for anonymization purposes because of rare diseases. We will not optimize this parameter.
3. **Window size (1-10)**
  Describes how many of the neighboring codes will be considered in each training step within the 2Vec algorithm and a given sequence of codes.
4. **Downsampling**
  Smaller values of the downsampling parameter mean that more of the most common words will be randomly excluded from the training data (default 0.001). After preliminary analysis, we observed that downsampling is always detrimental to our task, so we did not downsample our data.
5. **Epochs (1-20)**
  The number of training epochs describes how many times each patient’s code sequence will be looked at to update the vectorization model.
6. **Negative sampling (0-20)**
  For each update of a word and its neighboring words (within the window size range), this gives the number of random words not within the window that will be updated as negative examples; 0 for no negative sampling.
7. **Negative sampling exponent (–5 to 5)**
  Smoothing exponent for the updates of the negative samples.
8. **Hierarchical softmax (Boolean)**
  This parameter describes how the network parameters will be updated at the end of each training step; true for hierarchical softmax and false for no hierarchical softmax.
9. **Distributed memory or distributed bag of words (Boolean)**
  Training of document vectors in either distributed memory (DM) or distributed bag of words (DBOW) fashion (see above); true for DM and false for DBOW.
10. **Alpha (0.001-0.1)**
  Learning rate of the neural network updates.

### Regression and Classification Methods

#### Overview

The data matrices generated by binary encoding or Pat2Vec served as input data for prediction algorithms on the 4 calibration outcomes (number of cases, emergency health care utilization, age, and gender). The employed algorithms are described below, where LightGBM refers to the light gradient-boosted machine algorithm [50].

#### Regression

For the real-valued count outcomes of age and number of cases, we employed 2 different regression techniques: linear regression and an ensemble decision tree–based regression algorithm with gradient boosting (LightGBM Regressor) [50-52]. We chose LightGBM over other gradient-boosted tree methods because of its performance and fast training time [50,53,54]. Linear regression does not have additional input parameters; LightGBM was used out of the box without parameter optimization. The goodness of fit was measured by the *R*^2^ and 1 minus the relative mean absolute error (also known as Cumming predictive measure [CPM]) [55].

#### Classification

For the binary outcomes of gender and emergency usage, we employed 2 different classification techniques: logistic regression and an ensemble decision tree–based classification algorithm with gradient boosting (LightGBM Classifier) [50,52,56]. Logistic regression does not have additional input parameters; LightGBM was used out of the box without parameter optimization. The goodness of fit was measured by the area under the receiver operating characteristic curve and the area under the precision-recall curve.

### Final Model

The final model was chosen with Bayesian optimization of the hyperparameters by aggregating the 16 performance measures: 2 approaches with linear/logistic regression and gradient-boosted trees, and 2 measures for each of the 4 outcomes (*R*^2^ and CPM for regression, receiver operating characteristic curve and area under the precision-recall curve for classification). All of these measures are in the range of 0 and 1, with higher values indicating better performance but varying in size and range between the 4 different outcomes and measures. As such, we took the performance measure values of the top 100 diagnoses baseline model as reference values. For each trial in the Bayesian optimization and its respective vectorization model, we calculated the 16 performance measures and divided them by the respective reference value from the top 100 diagnoses baseline model. We then aggregated these rates by calculating their arithmetic mean as a total score (ie, this gives a reference score of 1 for the top 100 diagnoses baseline model). The final model was chosen based on the best total score after this aggregation (Figure 1).

We then trained embedding models with the same hyperparameter configuration as the final model, but with different vector sizes M. Likewise, we derived the binary encoding matrices of the top M diagnoses for varying sizes of M. These embedding and binarization models were compared on the same prediction tasks described above on the holdout test data. The same procedures were replicated on the different data sets for robustness analysis (diagnosis dropout and reduced training data size, respectively).

Additionally, we conducted an exploratory and visual analysis of the vector embeddings from the Pat2Vec vectorization on the test data. To this end, we projected the 100D patient vector embeddings into 2 dimensions using the uniform manifold approximation and projection (UMAP) algorithm [57]. In addition, these projections were clustered using hierarchical density–based clustering (hierarchical density–based spatial clustering of applications with noise [HDBSCAN]) [58]. We assessed the general demographic and health care properties of the clusters and identified overexpressed ICD-10 codes within each cluster as the codes that have the largest positive difference in their share within the respective cluster compared with their share in the general population. As an explainability analysis, we analyzed how ICD-10 diagnosis codes are associated with specific dimensions of the vector embedding of size 100. To this end, we calculated correlations over all patients in the test data between a subset of 60 relevant ICD-10 diagnosis codes, binary encoded per patient, and the 100 vector dimensions.

Furthermore, we predicted drug spending costs using the final embedding model with a vector size of 100 and the baseline model. We compared the performance (*R*^2^, mean absolute error, and CPM), again with linear regression and the gradient-boosted trees algorithm for regression (LightGBM Regressor). We also added age and gender as additional predictors to these models. Here, we tuned the hyperparameters of the LightGBM method using Bayesian optimization to achieve its full potential.

### Software

Analysis was conducted primarily in the Python programming language (Python Software Foundation) [59], with additional analyses in the R statistical programming language (The R Foundation) [60]. Pat2Vec was implemented using the Gensim package [61] for Python with hyperparameter tuning via the Optuna package [62]. Machine learning prediction tasks were conducted with scikit-learn (linear and logistic regression, [63]) and the LightGBM Python package [50], while 2D projection and clustering were based on the UMAP package [57] and the HDBSCAN package [58], respectively. Final visualizations were prepared in R with the ggplot2 package [64].

## Results

### Sample Characteristics

After filtering the original sample of 11,200,000 patients, the data were limited to 9,937,919 patients. The average age of the patients was 45.2 years; 54.60% (5,426,481/9,937,919) of the cohort were female. The average number of cases per patient in 2019 was 8.4. About 18.32% (1,820,736/9,937,919) of the cohort had at least one emergency in 2019. The average drug spending in 2019 was €632.1 (US $683.4). The average number of diagnosis codes from 2016 to 2018 (relevant for the training data) was 67.6, whereas the average number of codes in 2018 only (relevant for prediction tasks) was 34.6. Variance was very high on the variable drug spending, with an SD of 4383.9 (Table 1). Furthermore, we observed a high number of patients with a 0 value in drug spending in 2019 (2,132,938/9,937,919, 21.46%, patients).

**Table 1:** Patients’ data characteristics.

Characteristics	Values
Age (years), mean (SD)	45.2 (24.1)
Female gender, n/N (%)	5,426,481/9,937,919 (54.60)
Number of cases, mean (SD)	8.4 (6.7)
Emergency in 2019, n/N (%)	1,820,736/9,937,919 (18.32)
Drug cost (€^a^), mean (SD)	632.1 (4383.9)
Number of codes from 2016-2018, mean (SD)	67.6 (92.4)
Number of codes in 2018, mean (SD)	34.6 (45.5)

^a^€1=US $1.08 (as of March 27, 2023).

### Top M Diagnosis Codes

The baseline model was constructed from a binary encoding of the top M diagnosis codes, for varying numbers of M. The most prevalent diagnosis code was I10.90 (hypertension; 2,591,336/9,937,919, 26.08%, patients), followed by J06.9 (unspecified acute upper respiratory infection) and Z12.9 (unspecified special screening for neoplasms used in the various German cancer screening programs [65]). Many patients have at least one of the top diagnoses (eg, 8,947,182/9,937,919, 90.03%, patients) have at least one of the most prevalent diagnoses). By contrast, over 2000 unique diagnosis codes make up the bulk of the diagnoses, with a share of over 90% of all diagnosis codes (317,316,756/343,751,225, 92.31%) in the data (Supplementary Table S2 in Multimedia Appendix 1).

### Hyperparameter Tuning Results

**Figure 2:**
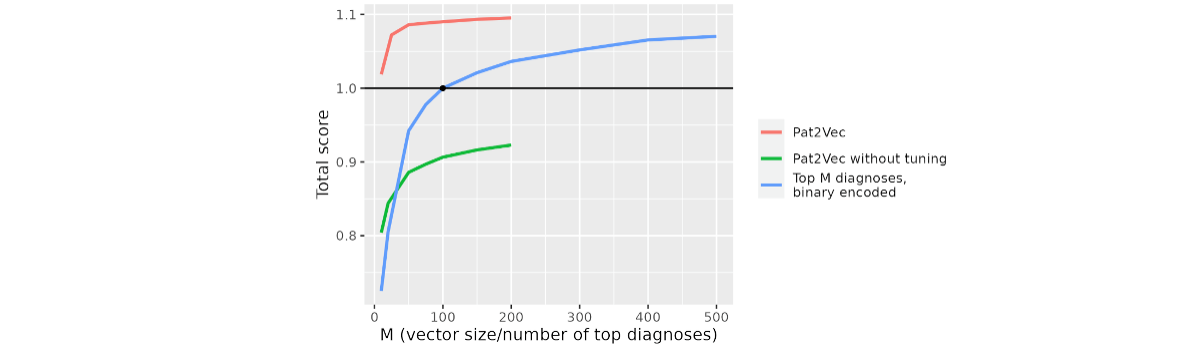
A comparison of the default vectorization model, the baseline model (the top M diagnoses), and the final model after hyperparameter tuning based on the total score of how well they did on prediction tasks.

The Bayesian optimization search for the best hyperparameter configuration revealed that the default parameters are not sufficient and can be greatly improved upon (Figure 2). The performance of the default parameter configuration did not exceed that of the top M diagnoses baseline model.

The most important hyperparameters (Supplementary Figure S1 in Multimedia Appendix 1) were (in order): the choice of DBOW over DM, the number of epochs (choosing 3), the negative sampling exponent (choosing approximately –2.3, compared with the default [0.75]), and the learning rate alpha (choosing approximately 0.0014, compared with the default [0.025]).

When compared with the top M diagnoses approach with M=100, the final set of parameters with a vector size of 100 resulted in a 9 percent point increase on our aggregated performance metric. All final models with a vector size of 10 or larger increased performance over this baseline model of the top 100 diagnoses. For smaller vector sizes, the gains in performance compared with the baseline models of equal size were larger (Figure 2). After a vector size of about 50, the performance of the vectorization increased by lesser amounts.

### Linear/Logistic Regression Versus Gradient-Boosted Trees

The ensemble-based machine learning with LightGBM Regressor/Classifier on the final vectorization model performed better than the linear and logistic regression counterparts on the vectorization data as well as the top M diagnoses data (Supplementary Figure S2 in Multimedia Appendix 1). Additionally, we observed a bigger increase in performance by switching from top M diagnoses data to Pat2Vec-derived vectors on smaller vector sizes, which stresses that information is compressed well by the vectorization. Furthermore, up to a vector size of about 100, the vectorization data with linear/logistic regression or LightGBM outperformed even the LightGBM approach on the binary-encoded data, which indicates that nonlinear properties of the patient profiles were encoded in the vector embeddings. In summary, using gradient-boosted trees or vector embeddings is always beneficial, and the combination of the 2 yields the best results.

### Robustness Analysis

#### Diagnosis Dropout

As a sensitivity or robustness analysis of the vector embedding (and the baseline binary encoding), we calculated total scores on the reduced dropout data (with 10%, 25%, and 50% of diagnosis codes missing, respectively). We observed a steeper decrease for the binary-encoded top 100 diagnoses data, while the performance of the vectorization suffers mildly even with a 50% drop out of the diagnosis data (Supplementary Figure S3 in Multimedia Appendix 1).

#### Vectorization Training Data Sample Size

As an additional robustness analysis of the vector embedding with regard to necessary training data size, we calculated total scores on reduced vectorization training data, from 100% (the original 10 million patients’ training data) to 0.1% of the original training data, or 10,000 patients. We observed a total score above 1 (thus, above the performance of the binary-encoded baseline model) for sample sizes as low as 0.5% of the original data, or 50,000 patients (Supplementary Figure S4 in Multimedia Appendix 1), while sample sizes of at least 1 million patients are needed to achieve total scores close to the total score on the original data.

### Analysis of Patient Embedding

**Figure 3:**
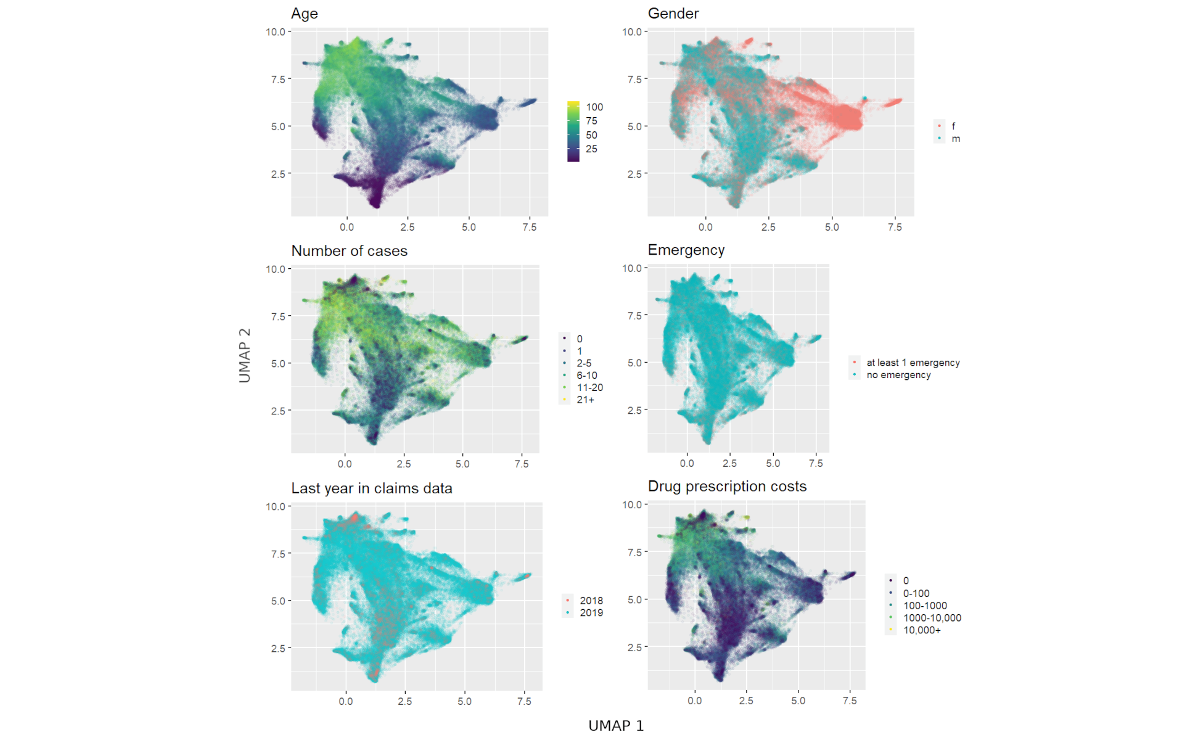
UMAP embedding of Pat2Vec, colored by age/gender/number of cases in 2019/emergency treatment in 2019/last available year in claims data/drug prescription costs in 2019. f: female; m: male; UMAP: uniform manifold approximation and projection.

**Figure 4:**
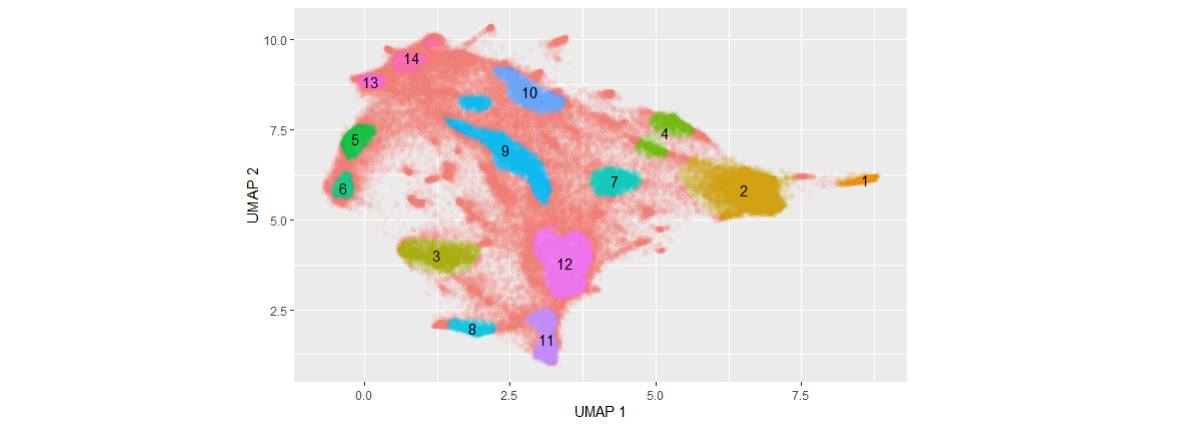
UMAP embedding of Pat2Vec, numbers 1-14 indicate clusters found by HDBSCAN (hierarchical density–based spatial clustering of applications with noise). UMAP: uniform manifold approximation and projection.

For visualization purposes, we projected the final vectorization model with a vector size of 100 into 2 dimensions using the UMAP algorithm. This way we were able to illustrate the high-dimensional vectorization and patterns within the patients’ cohort (Figures 3 and 4).

We observed a triangular shape in the vector space of the embedded patient profiles, with multiple regions of higher density. The 3 corner areas are (1) young patients of both genders with a low number of cases and low prescription costs; (2) women with an average age below the average age of the cohort and with low prescription costs and a medium number of cases; and (3) elderly patients of both genders with a high number of cases and high prescription costs (Figure 3). The HDBSCAN clustering identified 14 clusters but showed that many patients are not easily mapped to a cluster (50.67%, 42,024/82,937, of test data; Figure 4).

A closer inspection of the clusters revealed interesting patterns in the subcohorts (Figure 4 and Table 2; also see Multimedia Appendix 2 for further details). The clusters 5, 13, and 14 all have a mean age of almost 70 years or older, but differ in the share of females, mean number of cases, rate of emergency cases, and drug spending costs. Among these clusters, cluster 13 is the oldest with distinctive ICD-10 diagnoses of F03 (dementia) and R32 (urinary incontinence), along with a large number of patients who do not appear in 2019’s data, which indicates a high mortality within cluster 13. Clusters 5 and 6 have the most distinctive diagnosis codes in the H52 section (refractive errors/eyesight), but differ in their average age. Clusters 1 and 2 are almost exclusively female and of around the same mean age, but cluster 1 has a higher share of emergencies, and overexpressed ICD code Z34 (supervision of normal pregnancy) and section O09 (duration of pregnancy) point to pregnancy. Clusters 11 and 8 are the 2 youngest clusters, where cluster 11 is mostly characterized by routine examinations and vaccinations (Z00.1: routine child health examination; Z23.8 and Z27.8: immunizations), whereas cluster 8 is characterized by developmental disorders of speech and language (F80.9 and F80.0). Patients in cluster 12 have the most common acute ambulatory diseases (J06.9: acute upper respiratory infection; A09.9: gastroenteritis/colitis; and R51: headache). The remaining clusters show the other most prominent public health concerns in the German ambulatory health care system: cluster 3 (hay fever/asthma), cluster 4 (hypothyroidism), cluster 7 (depressive disorders), cluster 9 (pinched nerve/back pain/disc disorders), and cluster 10 (diabetes type 2).

Regarding the explainability or backward interpretation of our embedding, we analyzed how specific ICD-10 diagnosis codes map onto the patient vector dimensions. A heatmap of the correlations between a subset of 60 diagnosis codes and the 100D embedding showed that similar disease concepts were mapped to the same vector dimensions in a blockwise manner (Supplementary Figure S5 in Multimedia Appendix 1). It also showed that disease information was spread out over multiple dimensions instead of being mapped to only 1 dimension as in binary encoding.

**Table 2:** Properties of clustered patients’ cohorts.

Cluster	Percentage of cohort	Mean age (years)	Female, %	Mean number of cases	Emergency, %	Mean drug spending (€^a^)	Distinctive ICD-10^b^ codes
11	3.8	4.1	50.4	4.8	35.2	69.26	Z00.1, Z23.8, Z27.8
8	1.5	9.4	35.9	5.7	27.1	198.01	F80.9, F80.0, Z00.1
6	1.1	21.7	49.0	5.3	21.8	62.77	H52.2, H52.0, H52.1
12	6.7	27.6	31.3	4.6	19.8	175.77	J06.9, A09.9, R51
1	1.7	32.0	99.9	8.4	28.4	230.47	Z34, N89.8, O09.3
3	4.0	33.3	38.1	7.1	19.1	323.30	J30.1, J45.9, J45.0
2	9.3	33.7	99.7	8.6	18.7	130.00	N89.8, Z30.9, Z12.9
7	2.6	44.5	57.1	9.9	19.0	431.01	F32.9, F32.1, F33.1
4	2.4	48.6	86.7	9.9	13.9	191.26	E03.9, E06.3, Z12.9
9	6.6	57.6	47.0	10.4	15.7	592.98	M54.1, M51.2, M54.5
10	3.7	59.3	37.3	8.4	11.5	480.11	I10.9, I10.90, E11.9
5	2.1	69.9	59.6	10.9	12.9	809.16	H52.2, H52.4, H52.0
14	2.6	74.4	37.4	11.9	16.0	1587.98	I10.9, I10.90, I25.1
13	1.3	80.7	62.9	8.2	26.6	1248.64	F03, R32, I10.9
None	50.7	50.2	51.0	9.4	17.9	908.89	N/A^c^
All	100.0	45.6	54.5	8.7	18.7	654.17	N/A

^a^€1=US $1.08 (as of March 27, 2023).

^b^ICD-10: International Statistical Classification of Diseases and Related Health Problems, 10th Revision.

^c^N/A: not applicable.

### Prediction of Drug Spending Costs

Predicting prospective individual drug spending from diagnosis data is an especially hard task [66]. We predicted 2019’s patient-level drug spending based on patients’ diagnosis codes from 2018. We used and compared the binary-encoded top 100 diagnoses and our vectorization of dimension 100 (Pat2Vec). In addition, we extended the data by age and gender of patients. Table 3 shows the results using linear regression as well as gradient-boosted trees. We observed an overall high relative increase in performance by using the vectorization over the baseline model, while in general the *R*^2^ values were low. The linear regression shows diverging results between the top 100 and vectorization data with regard to absolute errors and squared errors (CPM and *R*^2^). The gradient-boosted trees approach to regression performed similarly to the linear regression on the baseline model of binary-encoded top 100 diagnoses, while the combination of Pat2Vec and gradient-boosted trees performed best. Adding age and gender as additional variables led only to small increases in performance.

**Table 3:** *R*^2^, mean absolute error, and Cumming prediction measure of predicting drug spending costs using linear regression and LightGBM Regressor.

Measure	Linear regression				LightGBM Regressor		
	*R*^2^, %	Mean absolute error (€^a^)	Cumming prediction measure, %	*R*^2^, %		Mean absolute error (€)	Cumming prediction measure, %
Age + gender	1.0	818.44	7.4	1.1		801.09	9.4
Top 100	2.0	760.55	14.0	2.1		755.76	14.5
Top 100 + age + gender	2.0	757.13	14.4	2.4		752.78	14.9
Pat2Vec	7.7	845.99	4.3	12.9		704.01	20.4
Pat2Vec + age + gender	7.7	845.98	4.3	13.7		690.70	21.9

^a^€1=US $1.08 (as of March 27, 2023).

## Discussion

### Principal Findings

We found that the NLP-based vector embeddings of claims data led to large improvements on health care–related prediction tasks compared with standard approaches (represented by binary encoding). Hyperparameter tuning is necessary for these improvements. On health care prediction tasks, gradient-boosted tree algorithms outperform standard statistical methods (linear or logistic regression). Gradient-boosted trees benefit more from vectorization. Additionally, the performance of the vectorization is more robust against incomplete data, but at least 1 million patients are needed to train the vectorization model. Furthermore, our cohort analysis shows that most patients’ diagnosis profiles lie on a spectrum of morbidity and cannot be easily mapped to distinct patient clusters. Overall, the results suggest we achieved the intended compression of the complete patient profiles while keeping the relevant amount of available information for prediction tasks.

### Comparison With Previous Research

Embeddings of diagnosis codes have been studied extensively before [14,29,38-42]. Patient-level embeddings have been derived rarely [14,25,42]. To the best of our knowledge, there is no ICD-10–based patient vectorization model trained and optimized for application in generalized health care tasks.

Choi et al [39] trained ICD-9 code representations using another similar NLP approach, and at the same time they learned “visit representations” (vectors) based on a binary encoding of the diagnosis codes for individual visits. Using logistic regression and these representations of visits, they were able to predict future disease codes from 1 visit to the next and clinical risk groups [27]. In a similar way, Pham et al [41] trained diagnosis code representations and combined them into variable-size “admission representations” as input for a long short-term memory (LSTM) to predict individual health prognoses after a health care intervention.

Miotto et al [25] derived a patient-level embedding (Deep Patient) using autoencoders based on ICD-9 diagnosis codes in conjunction with medications, procedures, laboratory tests, clinical notes (free-text), and demographic variables. They used random forests and patient embeddings to predict future diseases, but they did not tune their embedding algorithm or prepare it for more general tasks.

Nguyen et al [42] found diagnosis code embeddings using Word2Vec. Subsequently, given an outcome, they trained a convolutional neural network to find predictive motifs for a classifier. They arrived at a patient-level embedding after the convolutional neural network step, but these embeddings are dependent on the classification task (they predicted unplanned readmissions in a hospital setting).

Almog et al [14] applied a similar approach (Crystal Bone) to the special problem of predicting bone fracture incidents. For the prediction of this specific task, they trained their vectorization models on data filtered for bone incidents. They described 2 approaches: gradient-boosted trees (using XGBoost [67]) on patients’ vector embeddings as well as an LSTM [68] neural network on the individual sequences of patients’ diagnosis code embeddings. They observed better performance with the LSTM approach.

Li et al [29] derived an embedding for disease codes and a framework to predict diseases and even generalized outcomes (BEHRT). They did not set up a patient-level embedding with a fixed size, and their embedding framework needs to be retrained for new prediction tasks.

We were more interested in a general compression and embedding of patients themselves for general health care–related tasks (such as the prediction of different outcomes and an overall visualization) and not just the optimization of 1 prediction task only, thus we trained on the data of all patients, not filtered for specific diagnoses, and restricted ourselves to the analysis of the patients’ vector embeddings. In addition, our embedding is based solely on the ICD-10 diagnosis data and does not need additional data sources that might not be readily available in a claims data setting. It would be helpful to look into how well other advanced machine learning algorithms such as LSTM or convolutional neural networks work on the ICD or patient vector embeddings for health care prediction tasks, but this is outside the scope of this paper.

Adkins [69] discussed the implications of a widespread adoption of machine learning on EHR data in clinical prediction contexts. While arguing that more complex machine learning models (such as the one presented in this work, combining vectorization and ensemble trees) on growing bodies of data will yield more precise predictions at the price of interpretability (as well as unforeseen ethical and legal issues), they pointed out the limitations of considering a limited amount of ICD codes, a problem that we could address to a large extent in our work. Interpreting the dimensions of the vectorizations and other steps to “explainable machine learning/artificial intelligence” are still ongoing (eg, building on the Shapley additive explanations values for tree methods [70,71]). Here, we employed a simple approach using correlations between vector embeddings and binary encoding to allow interpretation of vector dimensions with regard to specific ICD-10 codes.

### Limitations and Strengths

It has been discussed that a fusion of EHR data (clinical/diagnosis data and laboratory quantitative measurements) and other data sources (eg, medical images and laboratory measurements) would lead to further advancements in health care prediction tasks [72,73], where the problems of these mixed data types need to be properly addressed. Unfortunately, the claims data of the presented analysis do not contain these additional data sources, and thus the current implementation cannot acknowledge this.

We set up access to a pretrained model of our vectorization with 10 dimensions so that other researchers in the field can evaluate our methods and use the model on their own health care data [74].

### Future Research

The next step will be to use the provided vectorization for relevant tasks to improve health care. We will investigate whether our approach will benefit tasks such as disease prediction with a long genesis time and prevention in cases of early detection, such as dementia and mild cognitive impairment. Furthermore, we will compare the benefits of data-driven vectorization with common EHR-based procedures such as the Elixhauser score [18] or clinical risk groups [27] in terms of describing patient cohorts or predicting health care outcomes. We think that patient clustering based on robust vectorization has the potential to identify patients who would benefit from early screening, which would lead to more personalized screening measures.

### Conclusions

Health care–related prediction tasks that rely on large samples of data should make use of vectorization instead of binary encoding. Our fully pretrained and validated model can be used on new and possibly small data sets as well. Advanced machine learning techniques profit more from our vectorization. We enable more precise prediction models for decisions on future public health policies as well as more accurate health care services for individual patients.

## Appendix

Multimedia Appendix 1 Information on previous studies, top M diagnoses, hyperparameter importance, performance comparisons, and vector loadings.

Multimedia Appendix 2 Extended Table 2 of main manuscript.

## Abbreviations

CPM: Cumming predictive measure
DBOW: distributed bag of words
DM: distributed memory
EHR: electronic health record
GDPR: General Data Protection Regulation
HDBSCAN: hierarchical density–based spatial clustering of applications with noise
ICD-10: International Statistical Classification of Diseases and Related Health Problems, 10th Revision
ICD-10-GM: International Statistical Classification of Diseases and Related Health Problems, 10th revision, German Modification
LightGBM: light gradient-boosted machine
LSTM: long short-term memory
NLP: natural language processing
SHI: statutory health insurance
UMAP: uniform manifold approximation and projection

## Data Availability

The data sets that formed the training data during this study are not publicly available due to the regulations for sensitive health data in Article 9 of the General Data Protection Regulation (GDPR) of the European Union. Access can be given by official boards within the context of specific research projects, and the authors are available to discuss such possibilities. An embedding model that was made as part of this study is available online so that other researchers in the field can evaluate our procedures and apply the model to their own health care data [74].
