# Supplementary material for: Patient Embeddings From Diagnosis Codes for Health Care Prediction Tasks: Pat2Vec Machine Learning Framework
Source: JMIR AI. 2023 Apr 21;2:e40755. doi: 10.2196/40755 (PMC11041498; doi:10.2196/40755)
Supplement: Multimedia Appendix 1 [file ai_v2i1e40755_app1.pdf]

Supplement (Patient Embeddings for Health Care Prediction Tasks: Pat2Vec Machine Learning Framework)

2023-04-19

1 Previous studies on embeddings with claims/diagnoses data

Table 1: Previous healthcare studies on embeddings with claims or diagnoses data. N patients refers to the number of patients in the embedding training data.

study	year	task	embedding of	N patients	country	data
Tran et al. [38]	2015	predict suicidal risk	diagnoses codes and procedures	7,578	Australia	diagnoses (ICD-10 codes), procedures, demographics
Miotto et al. [25]	2016	predict future diseases	individual patients	704,587	USA	diagnoses (ICD-9 codes), medications, procedures, lab tests, clinical concepts from free-text notes, demographics
Pham et al. [41]	2016	predict disease progression, interventions, risk of readmission	diagnosis codes and individual visits	4,794 (diabetes) / 4,073 (mental)	Australia	diagnoses (ICD-10 codes), medications, procedures
Choi, Y et al. [40]	2016	explore medical concepts	diagnosis codes, medications, procedures, lab tests	around 4,000,000	USA	diagnoses (ICD-9 codes), medications, procedures, lab tests
Choi, E et al. [39]	2016	predict future diseases codes and clinical risk groups	diagnosis codes and individual visits	440,271	USA	diagnoses (ICD-9 codes), medications, procedures
Nguyen et al. [42]	2017	predict clinical motifs and unplanned readmissions	diagnosis codes and procedures	around 300,000	Australia	diagnoses (ICD-10 codes), procedures
Almog et al. [14]	2020	predict future bone fractures	diagnosis codes and individual patients	490,221	USA	diagnoses (ICD-10 codes)
Li et al. [29]	2020	predict future diagnoses	diagnosis codes and individual patients	1,287,219	UK	diagnoses (Caliber codes), age

2 Top M diagnoses: reach within data

Table 2: Top M diagnoses and their reach within the data: percent of patients with at least one of the top M diagnoses, percent of all given diagnoses that are covered by the top M diagnoses codes.

M	patients	diagnoses
1	26.1	3.5
2	44.2	4.4
3	53.3	5.0
4	59.1	5.8
5	62.7	6.3
10	69.0	9.5
20	77.0	15.1
50	90.0	26.9
75	92.0	33.0
100	93.8	38.0
150	95.2	45.9
200	96.1	52.2
300	97.0	61.3
400	97.6	67.9
500	98.1	72.3
750	98.8	79.7
1000	99.1	84.4
2500	99.8	95.5
5000	100.0	99.1
10000	100.0	100.0

3 Hyperparameter importance

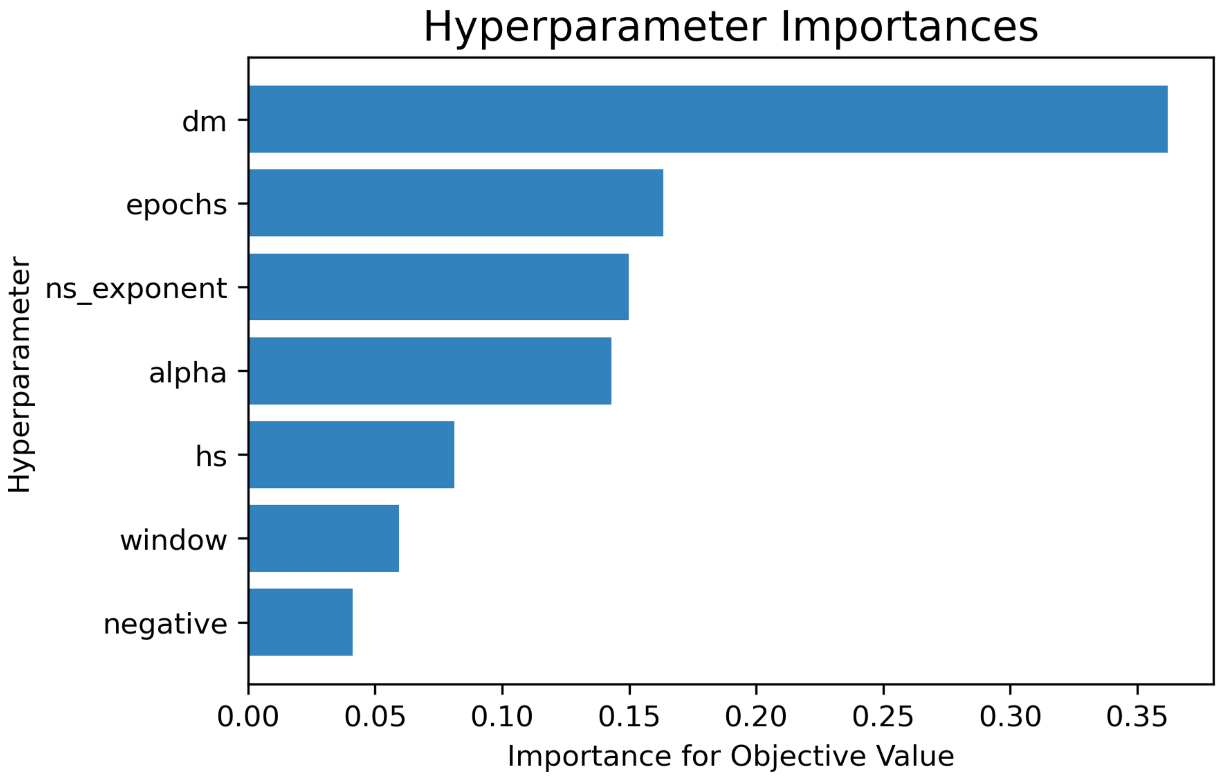

Figure 1: Optuna hyperparaneter importance.

4 Linear/logistic regression vs. gradient-boosted trees

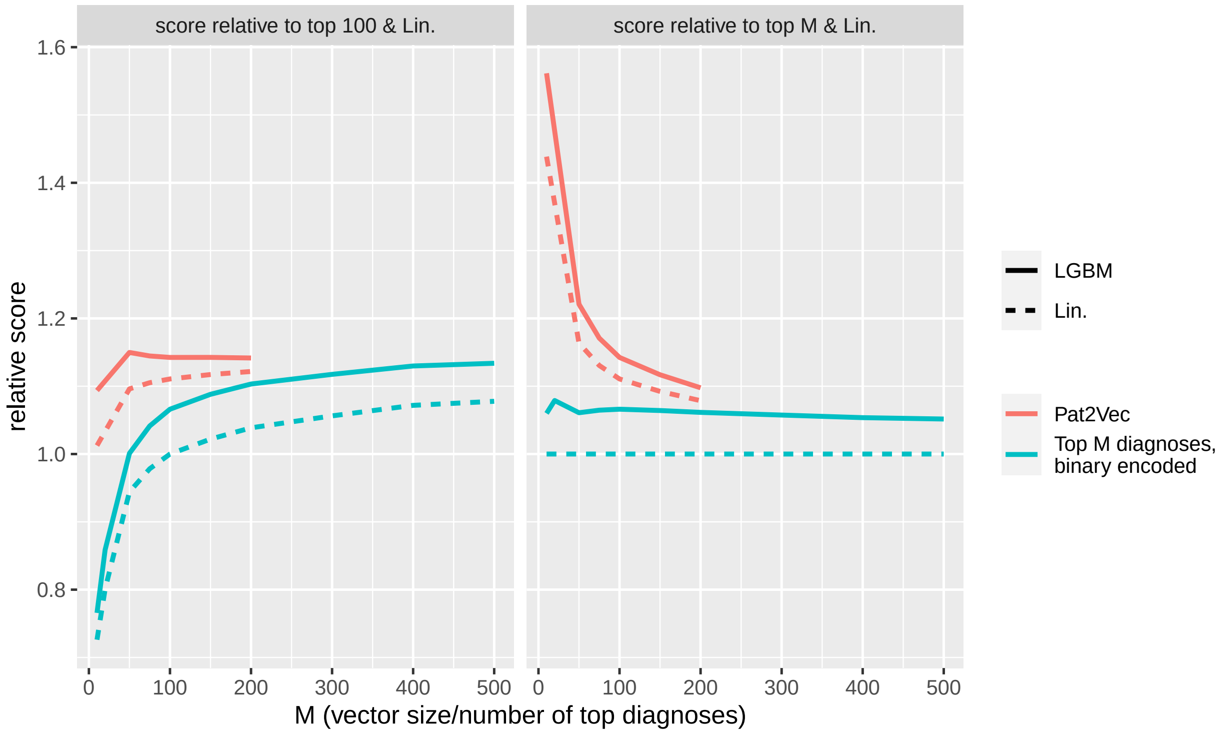

Figure 2: Performance of LGBM vs. linear methods. Left: The baseline is the top 100 diagnoses binary-encoded model with linear regression. Right: The baseline is the top M diagnoses binary-encoded model with linear regression.

5 Robustness: Diagnosis code dropout

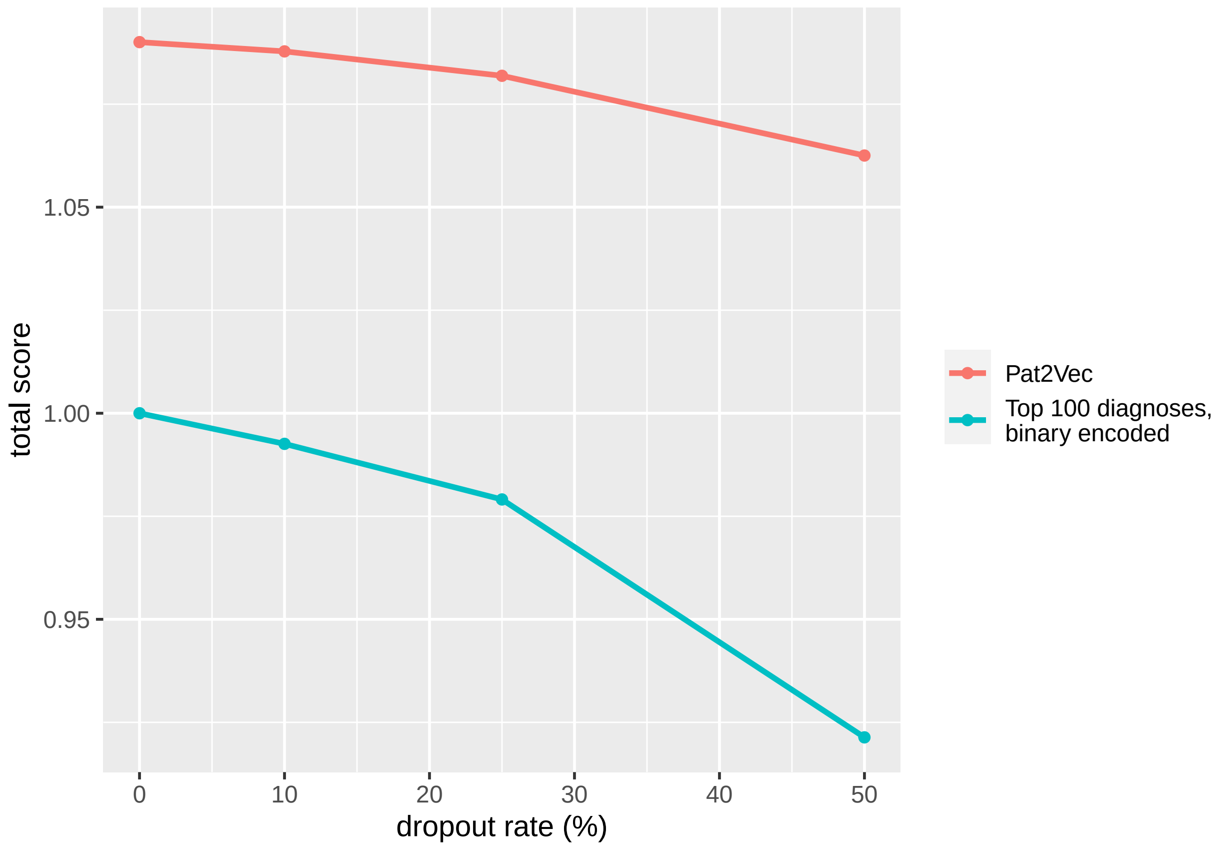

Figure 3: Robustness against dropout analysis: Comparison of the baseline model (top 100 diagnoses binary encoded) and the vectorization model on randomly reduced data (with 10 percent, 25 percent, or 50 percent dropout respectively).

6 Robustness: vectorization training data size

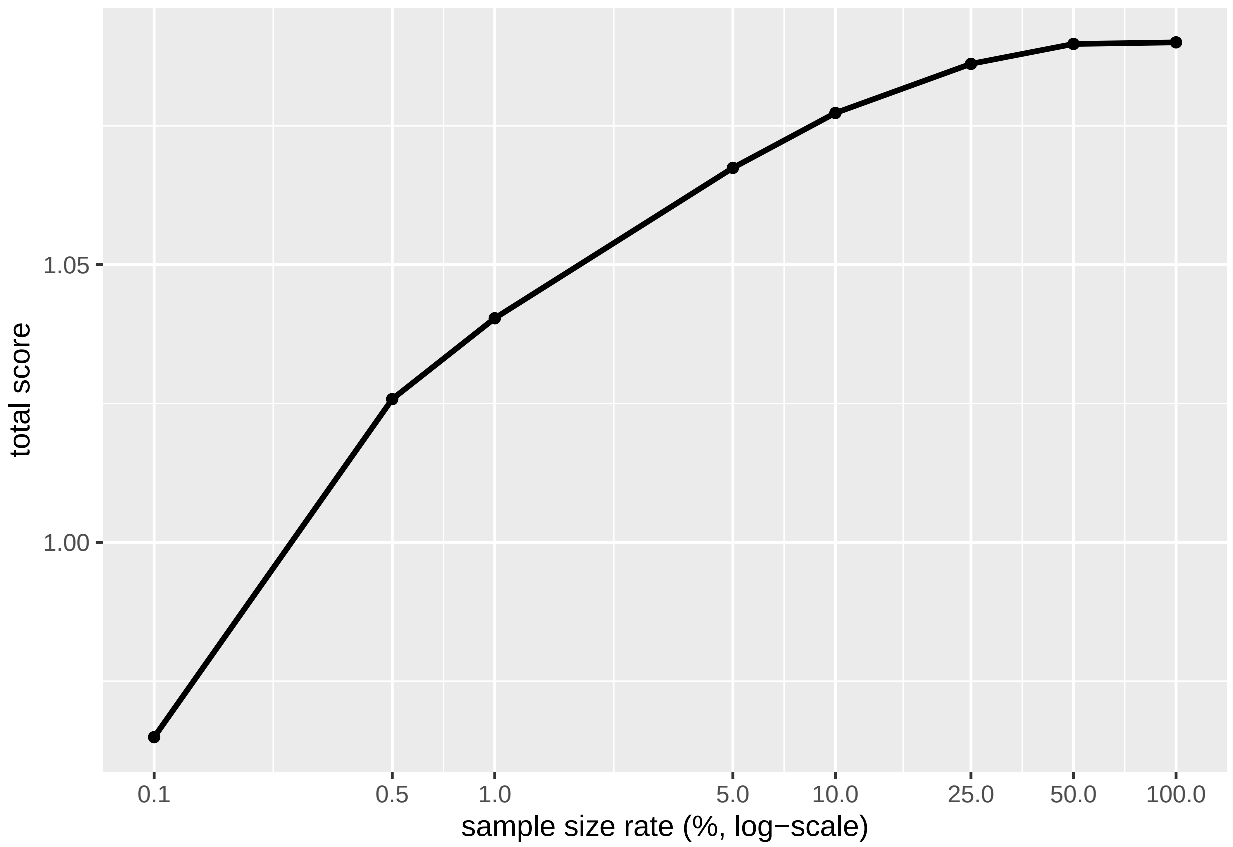

Figure 4: Analysis of sample size: Comparison of the baseline model (top 100 diagnoses binary encoded) and the vectorization model trained on different sample sizes.

7 Loadings of ICD-10 codes on vectorization embedding dimensions

We analyzed how a subset of ICD-10 diagnosis codes are associated with specific dimensions of the vector embedding of size 100. The chosen codes consist of the union of the top 20 diagnosis codes, all overexpressed ICD-10 codes from the cluster analysis (see main manuscript), and a handpicked sample of healthcare-relevant codes as well as some common rarer diseases. We calculated correlations over all patients in the test data between the binary encoded ICD-10 diagnosis codes and the 100 vector dimensions. Figure S3 shows a heatmap with hierarchical clustering/dendrograms of these correlations.

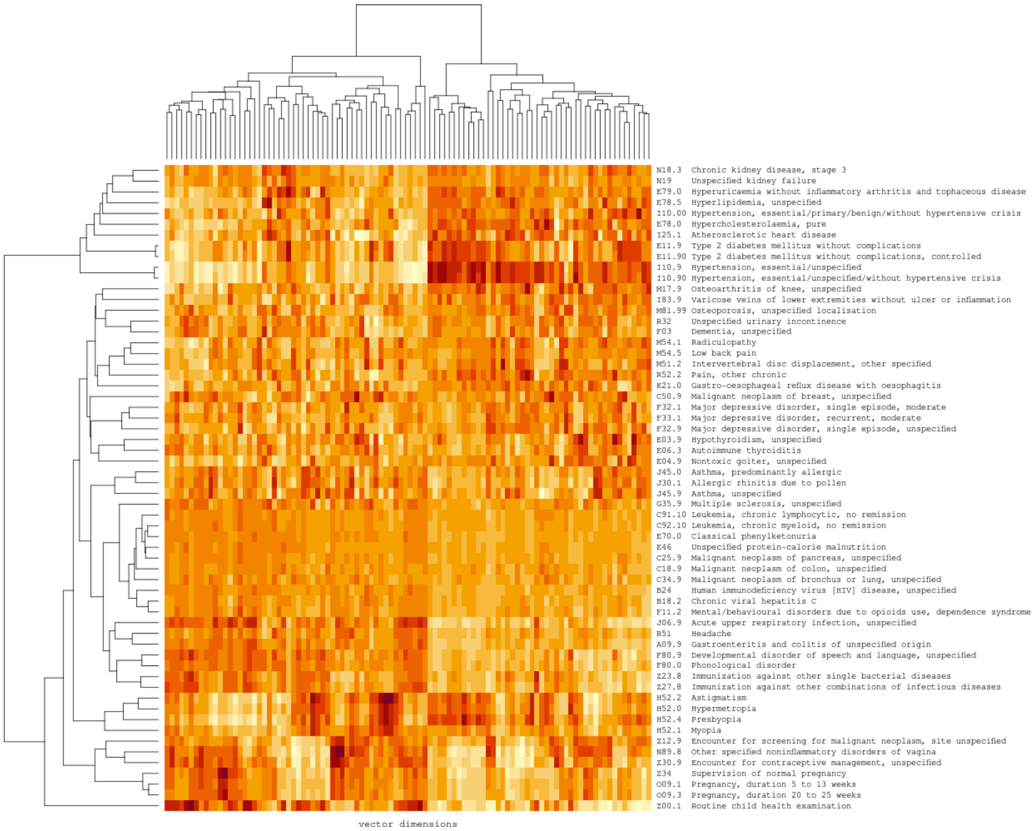

Figure 5: Heatmap of correlations between ICD-10 diagnosis codes and vector dimensions.

We observe meaningful clusterings of the diagnosis codes by similar disease concepts as well as highly similar correlation patterns for very similar identical codes (e.g. I10.9 and I10.90). Common codes map to specific blocks of dimensions, while rarer diseases have less pronounced patterns. We do not observe a distinctive pattern where every disease maps to a single dimension (like binary encoding), but rather the information spread over many dimensions. The original order of the dimensions is arbitrary and does not hold information due to random initialization of the embedding algorithm.

8 References

14. Almog YA, Rai A, Zhang P, Moulaison A, Powell R, Mishra A, et al. Deep Learning With Electronic Health Records for Short-Term Fracture Risk Identification: Crystal Bone Algorithm Development and Validation. J Med Internet Res 2020 Oct 16;22(10):e22550

25. Miotto R, Li L, Kidd B, Dudley J. Deep Patient: An Unsupervised Representation to Predict the Future of Patients from the Electronic Health Records. Sci Rep 2016 May 17;6:26094

29. Li Y, Rao S, Solares J, Hassaine A, Ramakrishnan R, Canoy D, et al. BEHRT: Transformer for Electronic Health Records. Sci Rep 2020 Apr 28;10(1):7155

38. Tran T, Nguyen TD, Phung D, Venkatesh S. Learning vector representation of medical objects via EMR-driven nonnegative restricted Boltzmann machines (eNRBM). J Biomed Inform 2015 Apr;54:96-105

39. Choi E, Bahadori M, Searles E, Coffey C, Thompson M, Bost J, et al. Multi-layer representation learning for medical concepts. New York, NY: Association for Computing Machinery; 2016 Presented at: 22nd ACM SIGKDD International Conference on Knowledge Discovery and Data Mining; August 13-17, 2016; San Francisco, CA p. 1495-1504.

40. Choi Y, Chiu CYI, Sontag D. Learning Low-Dimensional Representations of Medical Concepts. AMIA Jt Summits Transl Sci Proc 2016;2016:41-50

41. Pham T, Tran T, Phung D, Venkatesh S. DeepCare: a deep dynamic memory model for predictive medicine. In: Bailey J, Khan L, Washio T, Dobbie G, Huang JZ, Wang R, editors. Advances in Knowledge Discovery and Data Mining : 20th Pacific-Asia Conference, PAKDD 2016 Auckland, New Zealand, April 19–22, 2016 Proceedings, Part II. Cham, Switzerland: Springer; 2016:30-41.

42. Nguyen P, Tran T, Wickramasinghe N, Venkatesh S. A Convolutional Net for Medical Records. IEEE J Biomed Health Inform 2017 Jan;21(1):22-30.
