# Supplementary material for: Patient Embeddings From Diagnosis Codes for Health Care Prediction Tasks: Pat2Vec Machine Learning Framework
Source: JMIR AI. 2023 Apr 21;2:e40755. doi: 10.2196/40755 (PMC11041498; doi:10.2196/40755)
Supplement: Multimedia Appendix 2 [file ai_v2i1e40755_app2.pdf]

Supplement 2 (Patient Embeddings for Health Care Prediction Tasks: Pat2Vec Machine Learning Framework)

2023-04-19

1 Full Table 2 of Main Manuscript

Cluster	Size of cluster (%)	Mean age (years)	Female (%)	Mean number of cases	Emergency (%)	Mean drug spending (€)	Distinctive ICD-10 codes
11	3136/82,937 (3.8)	4.1	1582/3136 (50.4)	4.8	1103/3136 (35.2)	69.26	Z00.1, Z23.8, Z27.8
8	1256/82,937 (1.5)	9.4	451/1256 (35.9)	5.7	340/1256 (27.1)	198.01	F80.9, F80.0, Z00.1
6	894/82,937 (1.1)	21.7	438/894 (49.0)	5.3	194/894 (21.8)	62.77	H52.2, H52.0, H52.1
12	5541/82,937 (6.7)	27.6	1735/5541 (31.3)	4.6	1097/5541 (19.8)	175.77	J06.9, A09.9, R51
1	1379/82,937 (1.7)	32.0	1378/1379 (99.9)	8.4	392/1379 (28.4)	230.47	Z34, N89.8, O09.3
3	3288/82,937 (4.0)	33.3	1254/3288 (38.1)	7.1	629/3288 (19.1)	323.30	J30.1, J45.9, J45.0
2	7710/82,937 (9.3)	33.7	7688/7710 (99.7)	8.6	1440/7710 (18.7)	130.00	N89.8, Z30.9, Z12.9
7	2186/82,937 (2.6)	44.5	1249/2186 (57.1)	9.9	416/2186 (19.0)	431.01	F32.9, F32.1, F33.1
4	2006/82,937 (2.4)	48.6	1738/2006 (86.7)	9.9	279/2006 (13.9)	191.26	E03.9, E06.3, Z12.9
9	5465/82,937 (6.6)	57.6	2567/5465 (47.0)	10.4	861/5465 (15.7)	592.98	M54.1, M51.2, M54.5
10	3102/82,937 (3.7)	59.3	1157/3102 (37.3)	8.4	358/3102 (11.5)	480.11	I10.9, I10.90, E11.9
5	1782/82,937 (2.1)	69.9	1063/1782 (59.6)	10.9	229/1782 (12.9)	809.16	H52.2, H52.4, H52.0
14	2131/82,937 (2.6)	74.4	797/2131 (37.4)	11.9	342/2131 (16.0)	1587.98	I10.9, I10.90, I25.1
13	1048/82,937 (1.3)	80.7	659/1048 (62.9)	8.2	279/1048 (26.6)	1248.64	F03, R32, I10.9
none	42,013/82,937 (50.7)	50.2	21,407/42,013 (51.0)	9.4	7536/42,013 (17.9)	908.89	
all	82,937/82,937 (100.0)	45.6	45,163/82,937 (54.5)	8.7	15,495/82,937 (18.7)	654.17	
